# Challenges and Supports to Aging in Place in a Gentrifying Context

**DOI:** 10.1093/geroni/igaa057.3227

**Published:** 2020-12-16

**Authors:** Manish Kumar, Laura Richman

**Affiliations:** 1 Duke University, Durham, North Carolina, United States; 2 Duke University School of Medicine, Menlo Park, California, United States

## Abstract

Neighborhoods play a central role in healthy aging, with changes to neighborhoods having a profound impact on older adults’ ability to age in place. Using gentrification as an indicator of neighborhood change and applying the theoretical framework of the Environmental Press model (Lawton and Nahemow, 1973), this study examined the relationship between changing environments, affordable housing, and environmental attributes that support and hinder the health and well-being of older adults. A qualitative, case-study approach was used to interview low-income, majority Black older adults in a gentrifying area of Washington DC. 32 individuals (16 in non-profit and 16 in for-profit affordable housing) aged 55 and older participated in semi-structured interviews on perceptions of gentrification, neighborhood change, and challenges and supports to aging in place. Transcripts were then analyzed using the framework method of analysis. Although participants generally reported that gentrification improved their neighborhood’s built environment, many attributed it to a decline in social capital. Affordable housing provided an ability to age in place, though participants expressed uncertainty over their long-term ability to age in the context of continuing change. These findings suggest that while the physical changes accompanying gentrification may support older adults’ ability to age in place, its detrimental impact on social capital further increases their risk for social isolation. While affordable housing may enable older adults to age in place, fostering a greater sense of permanence and well-being will require additional policies that both increase accessibility to the physical amenities provided by gentrification and preserve older adults’ social capital.

